# *Making the invisible visible*: a place for utilizing activity theory within in situ simulation to drive healthcare organizational development?

**DOI:** 10.1186/s41077-020-00148-8

**Published:** 2020-10-23

**Authors:** Gerard J. Gormley, Anu Kajamaa, Richard L. Conn, Sarah O’Hare

**Affiliations:** 1 Centre for Medical Education, Queen’s University Belfast, Belfast, Northern Ireland; 2 Faculty of Educational Sciences, University of Helsinki, Helsinki, Finland

**Keywords:** General practice, Community-based healthcare, In situ simulation, Activity theory

## Abstract

**Background:**

The healthcare needs of our societies are continual changing and evolving. In order to meet these needs, healthcare provision has to be dynamic and reactive to provide the highest standards of safe care. Therefore, there is a continual need to generate new evidence and implement it within healthcare contexts. In recent times, in situ simulation has proven to have been an important educational modality to accelerate individuals’ and teams’ skills and adaptability to deliver care in local contexts. However, due to the increasing complexity of healthcare, including in community settings, an expanded theoretical informed view of in situ simulation is needed as a form of education that can drive organizational as well as individual learning.

**Main body:**

Cultural-historical activity theory (CHAT) provides us with analytical tools to recognize and analyse complex health care systems. Making visible the key elements of an in situ simulation process and their interconnections, CHAT facilitates development of a system-level view of needs of change.

**Conclusion:**

In this paper, we theorize how CHAT could help guide in situ simulation processes—to generate greater insights beyond the specific simulation context and bring about meaningful transformation of an organizational activity.

## Introduction

Adaptability and agility are crucial attributes for any progressive organization and no more so than in healthcare. With one of the largest workforces in the world, there is a continual need to generate and implement new practices into workplace contexts. This represents a major challenge not just for individual workers, but for educators, whose responsibility is to train the workforce. Increasingly, in situ simulation is being utilized as means of translating evidence in to real world practice. As this modality increases in its use in healthcare, there is a need to further enhance our understanding of in situ simulation and for it to be guided by theoretical models to optimize its impact.

Cultural-historical activity theory (CHAT) [1] is a theoretical framework that provides us with analytical tools to understand complex activities such as real world clinical practice—yet has gained little attention regarding in situ simulation. In this article, we will consider the role of in situ simulation, introduce the concept of CHAT and then theorize its potential to guide and inform in situ simulation and bring about organizational change.

### Simulation: *preparing persons, people and processes*

Simulation has come to the fore in preparing individuals, teams and systems for improved healthcare provision. In recent times, the COVID-19 pandemic has been an example of where simulation has been harnessed to best prepare our workforce to meet such challenges [2–4]. Simulation contrasts with other modalities of education such as online learning: as well as advancing intellectual skills, it enables knowledge to be translated into practice. Typically, simulation-based education occurs in locations remote from clinical environments (e.g. simulation centres). However, simulation can also occur in practitioners’ authentic working clinical environments—i.e. in situ simulation—for example in community-based healthcare facilities.

In situ simulation provides real world contextuality and allows individuals, and teams, authentic learning experiences in their place of daily work [5]. In other words, in situ simulation offers a form of education that can create, not just translate, knowledge, supporting a zone of development and enabling ‘learning of something that is not yet there’—i.e. expansive learning [1, 6]. This includes extending beyond limits of individual capability in a scaffolded, supportive and safe approach.

Existing literature provides evidence of how lessons learned from in situ simulations have been utilized to transform healthcare systems [5]. In doing so, new healthcare system pathways have been developed, and latent hazards identified and mitigated, for example, utilizing in situ simulation to evaluate the operational preparedness for treating patients with COVID-19 in intensive care units [7]. Yet, despite the educational potential of simulation processes, so far little research attention has been directed to the role of theoretical models in facilitating collective creation of knowledge and learning with in situ simulation.

### In-situ simulation in sscommunity healthcare contexts

Traditionally, in situ simulation has been hospital-centric [5]. However, in situ simulation has at least as much to offer within community settings, given their diverse range of environments and magnitude of clinical activity. As in all clinical environments, there is a constant need to be adaptive to meet the healthcare needs of our societies. For example during the COVID-19 pandemic, many community-based healthcare settings had to entirely re-orientate their services: forming community-based hubs to triage, assess and manage patients with possible COVID-19; providing testing centres for those with symptoms or contacts; and re-configuring services to provide safe care for those with pre-existing health needs [8].

By supporting collective creation of knowledge and learning, in situ simulation can help to develop and model new activities of care in community-based healthcare ecosystems such as these. Specifically, it can facilitate the key process of harmonizing policies into a diverse range of community-based clinical settings. Given the relative underutilization of in situ simulation in the community, however, potential users need to be guided in how best to introduce this form of simulation. To this end, there are an increasing number of models to assist in its implementation [9–11]. Many of these tools help educators identify the key elements of the healthcare systems that need to be analysed and transformed. This enables a shift in focus away from simulation’s traditional emphasis on skill development of individuals at a micro-level, towards the systemic elements of organizational activity. CHAT offers a particularly useful analytical approach for challenging and expanding this predominant view, given its focus on making visible the key elements of an organizational activity and the complex interrelationships and tensions between them [1]. In the next section, we will describe this theory and contextualize how CHAT has the potential to inform in situ simulation to transform a clinical activity.

### Educating for systemic change: *introducing activity theory*

At the heart of any theory is its ability to widen our understanding of phenomena. By drawing upon ideas and concepts, theory sets out to help us make sense of activities. For example, *cognitive load theory* is widely used in the domain of simulation [12]. In essence, this theory explains the concept that an individual’s *working memory* has a limited capacity when dealing with information. When this capacity is surpassed, performance can be impaired—such as cognitive distraction of a surgeon performing a laparoscopic procedure [13].

All theories aim to help us to conceptualize the elements and influences of phenomena, thereby allowing us to develop a critical understanding and design targeted interventions. When considering the phenomena of clinical work, there are a multitude of elements and influences that come into play. Aside from healthcare professionals, a bewildering range of factors contribute to processes of patient care. From available equipment, clinical guidelines that have to be adhered to, administrative staff who contribute to activity and the condition and perspectives of patients, the amount of variables and their relationships can appear endless. Characteristic of such authentic and complex human activities, work-related activities are, however, rarely linear and often appear unpredictable.

Yet, in CHAT, cognitive instruments, such as analytical models and concepts, are regarded as an essential part of joint collective human activity and as a way to understand the activity, to give it meaning and to develop it. These instruments must always be examined in relation to the context in which they are used [6, 14]. CHAT provides us with analytical tools, such as the theoretical-methodological model of an activity system to recognize and understand such complex systems [1]. The care of a single patient usually requires the involvement of many different professionals belonging to different activity systems conducting care activity and simultaneously producing complex [15].

**Fig. 1:**
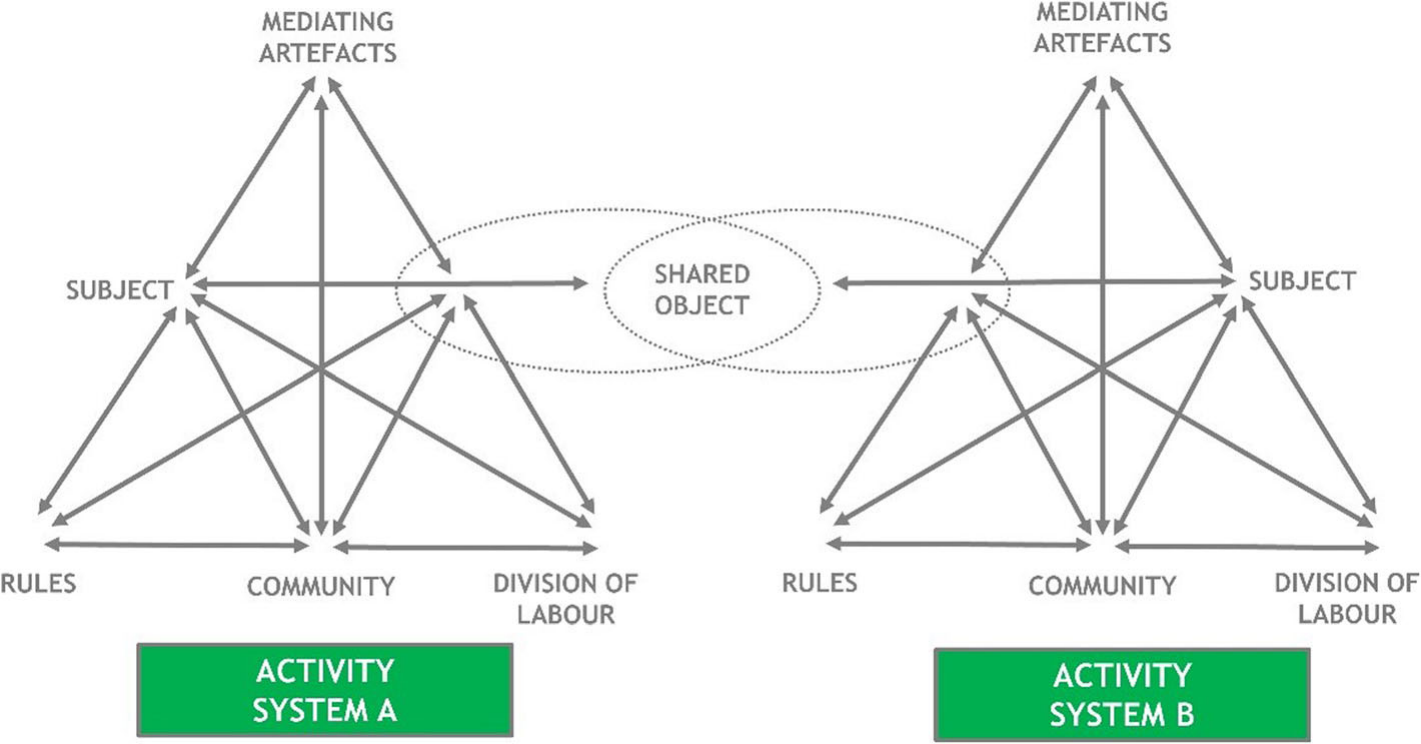
Activity as a dynamic model of interlinked activity systems (A and B) [1]

The historical analysis of collective work activities and collectively focused units of analysis are emphasized in activity-theoretical studies. Amongst CHAT, the structure of human activity is presented as a dynamic model of an activity system consisting of a *subject* (or group of subjects), an *object*, *mediating artefacts*, *rules*, a *community* and *the division of labour* (see Fig. 1). Every organization forms a system, which consists of activity systems and their objects. In organizations, activity systems exist in relation to neighbouring activities and their different objects of activity [1]. Subjects (e.g. medical practitioners) act as parts of a larger community of practitioners that performs object-oriented collective activity. The distinction between activity systems is made by their conception of the object (in health care the patient) [16]. Activity theory regards tools, division of labour and rules which mediate human activity as essential devices in organizational learning processes. Following the rules often means that the practitioners try to avoid mistakes and deviations from standardized protocols. Community building can lead to collectively created new activity models, responsibility and process efficiency [17].

Activity-theoretical methodology specially focuses on enhancing collective learning through the analysis of tensions and the reconceptualization and development of a shared object of the collective activity. Thus, many discussions of CHAT deal with the concept of an *object* [15, 18]. The identity of any activity is primarily determined by its *object* (generally, in the context of health care, the patient), which includes a collective motive for the activity. The historically established division of labour between levels of care and medical specialties outlines the object of activity. Object-oriented actions are always characterized by ambiguity, surprise and sense-making and include the potential for change, i.e. the expansion of the *object* [1]. Further, every organization forms a system, which consists of activity systems and their objects. In organizations, activity systems exist in relation to neighbouring activity systems and their different objects of activity. In this way, activity theory, with its interventionist orientation, represents an interactive form of social science [19]. Activity systems are inherently multi-voiced since the subjects form different conceptualizations of the object. The activity system model is designed to explore the tension-laden relationships between the elements within singular activity systems and between multiple interacting activity systems [1]. The system-level view transcends the view that focuses on certain parts of organizations, viewing an organization as a complex system of interacting elements and stakeholders, maintaining mutually exclusive structures, such as stability and instability [20]. The analytical focus needs to be on two or more entities, such as on the multiple voices mirroring different logics and the interaction between different professional groups [15]. Thus, studies analysing networks of activity systems have increased as activity-theoretical studies have started, over the last two decades, to focus on examining the interrelations and interactions amongst multiple activity systems.

### Using CHAT to target organizational learning through in situ simulation

To illustrate how CHAT can be utilized to inform and guide in situ simulation to facilitate organizational change, we will describe a community healthcare base theoretical example. However this example could also be considered in many other contexts such as secondary care.

### Theoretical case vignette

- Context of clinical activity

With the COVID-19 pandemic, a general practice needs to review and adapt its processes of managing patients, with suspected COVID-19, who have a cardiac arrest whilst on their premises. Given the infection risks associated with COVID-19, the general practice needs to remodel its process to ensure they maintain the highest standards of care and infection control. Patients with COVID-19 are at a greater risk of hypoxia [21]. Older patients and those with multi-morbidities are at a risk of high acuity events such as cardiac arrest [22]. Therefore, it is paramount that the general practice has systems in place to manage such critical and highly time dependent events. Importantly, donning appropriate PPE introduces a potential significant delaying factor in administering resuscitative interventions such as basic life support (BLS) [23]. In order for the practice to be best prepared for such events, the practice sets out to conduct an in situ simulation, guided and informed by CHAT, to contextually test their current systems and help target organizational transformation to improve their preparedness for such emergency situations.

Other theoretical examples could include an outpatient department or day procedure unit also wanting to review and advance their current systems of providing resuscitative care with enhanced infection control measures brought about by COVID-19.

- Preparing for an in situ simulation

The processes of in situ simulation have been well described elsewhere [9–11]—so we will provide only a few key points. Prior to conducting an in situ simulation, there are a number of preparatory steps required to be undertaken by the practice. Firstly, it is important to engage a diverse range of stakeholders—not only healthcare professionals but also the many others who contribute to the health system such as administrative staff, cleaning staff and practice management. Secondly, it is important to carry out a risk assessment to mitigate and harm brought about by the in situ simulation (for example, replacing real Automated External Defibrillators (AEDs) with training AEDs; take measures to monitor and control for any unexpected individuals who may witness the simulation. Such individuals will have not had a briefing and have a risk of being traumatized by the simulation). It is crucial that clinical service is not impacted. Therefore, a suitable time period will have to be identified. Lastly, any necessary simulation training materials will need to be obtained and placed in situ (e.g. a resuscitation manikin)

- Utilizing CHAT to conduct and guide in situ simulation

As in any in situ simulation, there are the important phases including *briefing* participants, *conducting* the actual simulation and *debriefing*. From the outset, it is important to be inclusive of all individuals who contribute to the clinical activity, i.e. the *subjects*. In the context of this clinical activity, the *subjects* of the activity are healthcare works (HCWs) involved in providing resuscitative care to a collapsed patient with COVID-19 whilst in, or near to, the general practice premises. In healthcare, there can be HCWs who may not immediately consider they contribute to certain clinical activities but using the grounding of CHAT—it encourages the team to consider the wider community of who to involve in the in situ simulation. For example administrative staff may initially consider they should not be involved in the simulation. However, they do and can play an important role in many ways—for example ringing for paramedic assistance and assisting in family liaison. Practice pharmacists can consider the vital drugs required in such emergencies and enhance the system to ensure they are update and replaced if expired.

Fundamental to this in situ simulation process is for all to have a shared understanding of the purpose of the exercise. CHAT emphasizes the importance of identifying the *object* of the activity system. In this theoretical clinical example, the overall objective is to improve the general practices’ readiness to provide effective and efficient BLS whilst maintaining the highest safety standards for all involved. Bringing clarity to the object of everyone’s efforts is of critical important and should be continually referred to throughout the in situ simulation process. Having a shared understanding of the *object* of the activity can hold the community together and gives it a purpose [18]. It therefore is important that all participants have time to construct, conceptualize and identify the (at least partially shared) *object* of the activity systems involved in patient care. Dialogue during the briefing and debriefing process will help promote participants’ shared sense making of this *object*.

During the in situ simulation *briefing*, participants can be introduced to the important analytical principles of CHAT, such as the activity system model, thereby helping them to make ‘visible’ the various key elements of the clinical activity and open up opportunities for improvement. During the actual in situ simulation, it would be desired for participants to enact their ‘real’ responses to the emergency and not necessarily keep the key elements of CHAT in foremost of their minds. However, observers of the simulation can be mindful not only of the actions of participants, but also the key elements of the activity system informed by CHAT.

**Fig. 2:**
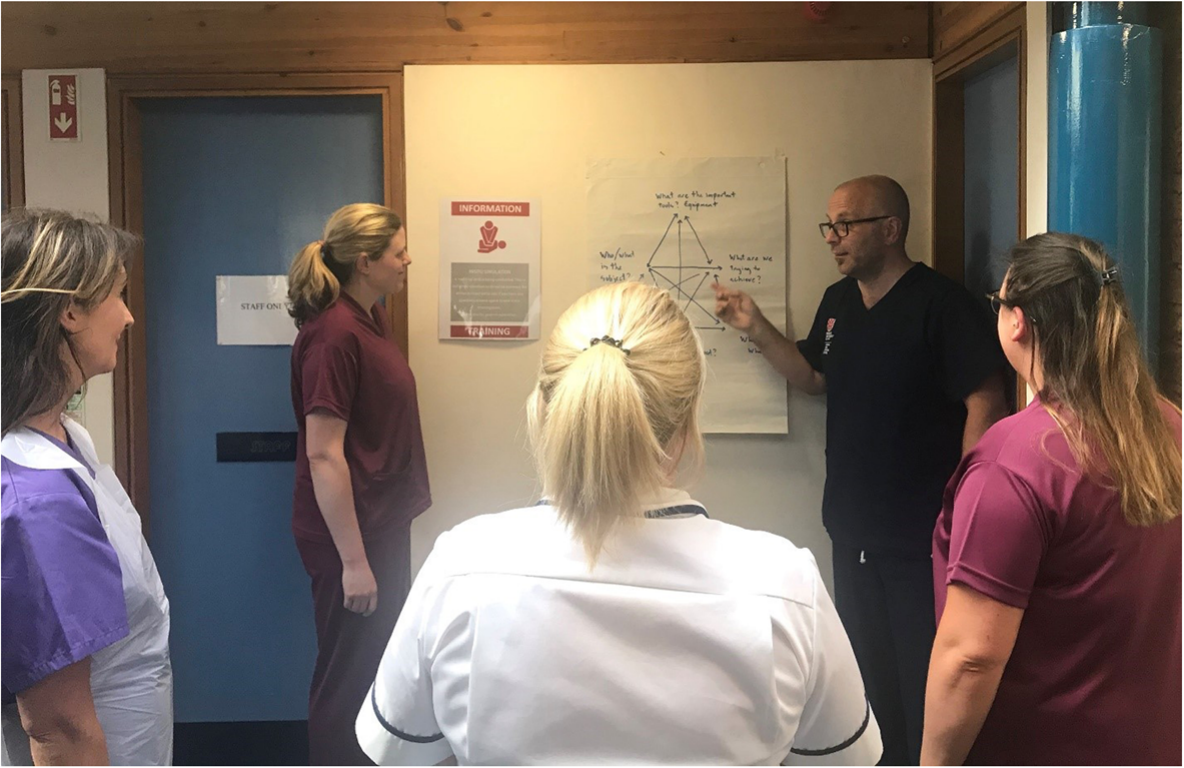
Illustration of an in situ simulation utilizing CHAT as a guiding focus during the briefing/debriefing

In the *debriefing* phase of the in situ simulation, it will be important for individuals to properly debrief their performance ensuring their psychological safety is maintained. Through the debriefing, it would be important for all stakeholders to collectively reflect on the activity as it happened and consider the various elements described above. By analysing the current system, a possibility for organizational development can emerge, enabling the community to improve their practice. The model of an activity system (see Fig. 2) described can be utilized to explore the tension-laden relationships within an activity system and between interconnected systems.

**Fig. 3:**
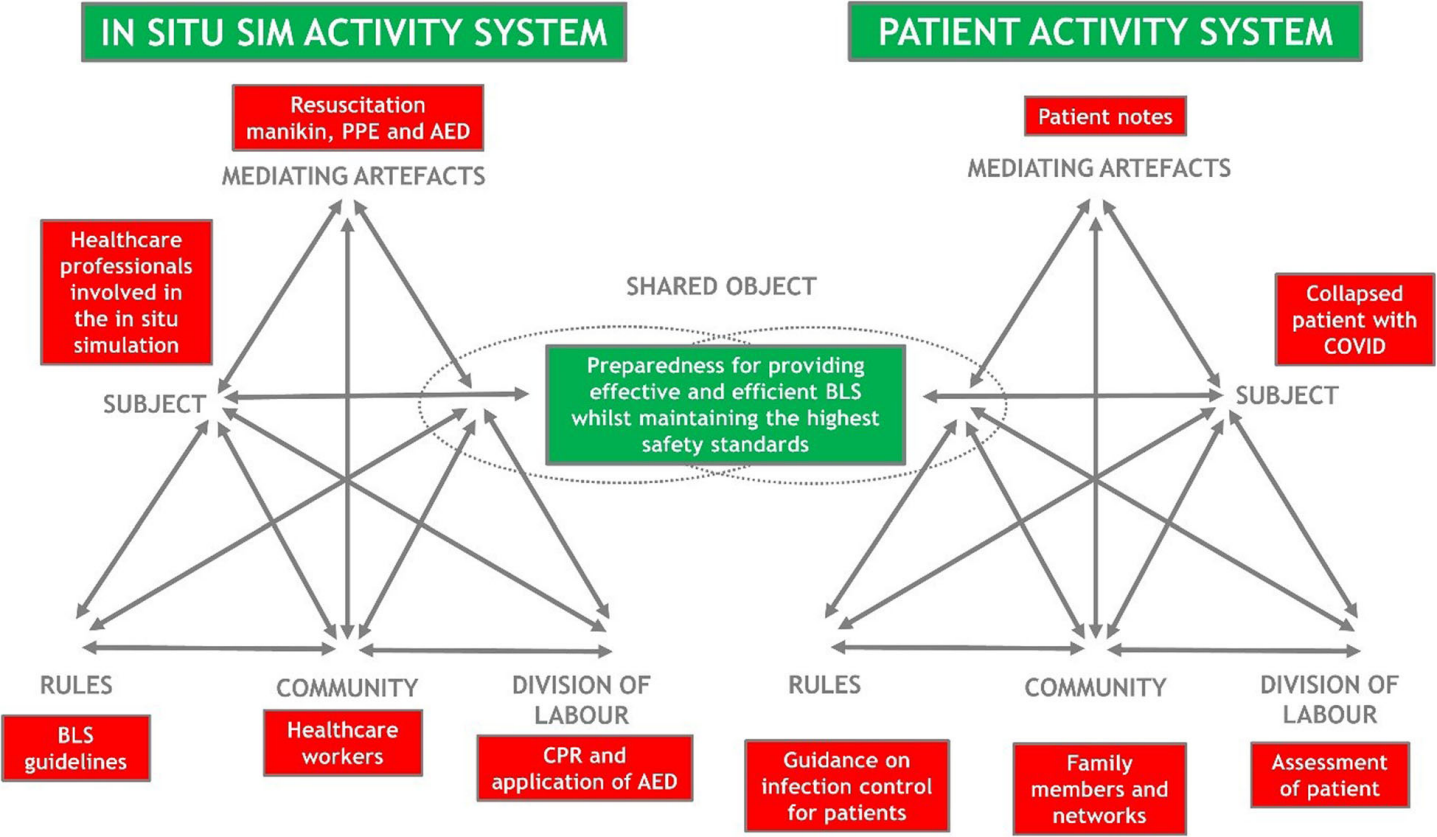
Diagrammatic representation of activity as a dynamic model of interlinked activity systems: in situ general practice-based simulation and that of a collapsed patient

Aside from the technical aspects of BLS, the elements of the activity system provide a wider view on the organization as a systemic whole. As the simulation unfolds, adaptions to the current system and entirely new forms of working may come to light. These may include introduction of new equipment by which efficiencies could be made, or revealing potential latent hazards. As a result of the renegotiation and reorganization of collaborative relations and practices, such tensions can be turned into drivers for change to overcome challenges and to facilitate organizational transformation, i.e. creating a zone of proximal development to allow for organizational change to emerge (see Fig. 3) [6].

We will now explore how using CHAT could provide and guide an analytical focus through this in situ simulation process and bring about transformational change in this theoretical case.

### Transformational changes brought about by CHAT informed in situ simulation

- Mediating artefacts

**Fig. 4:**
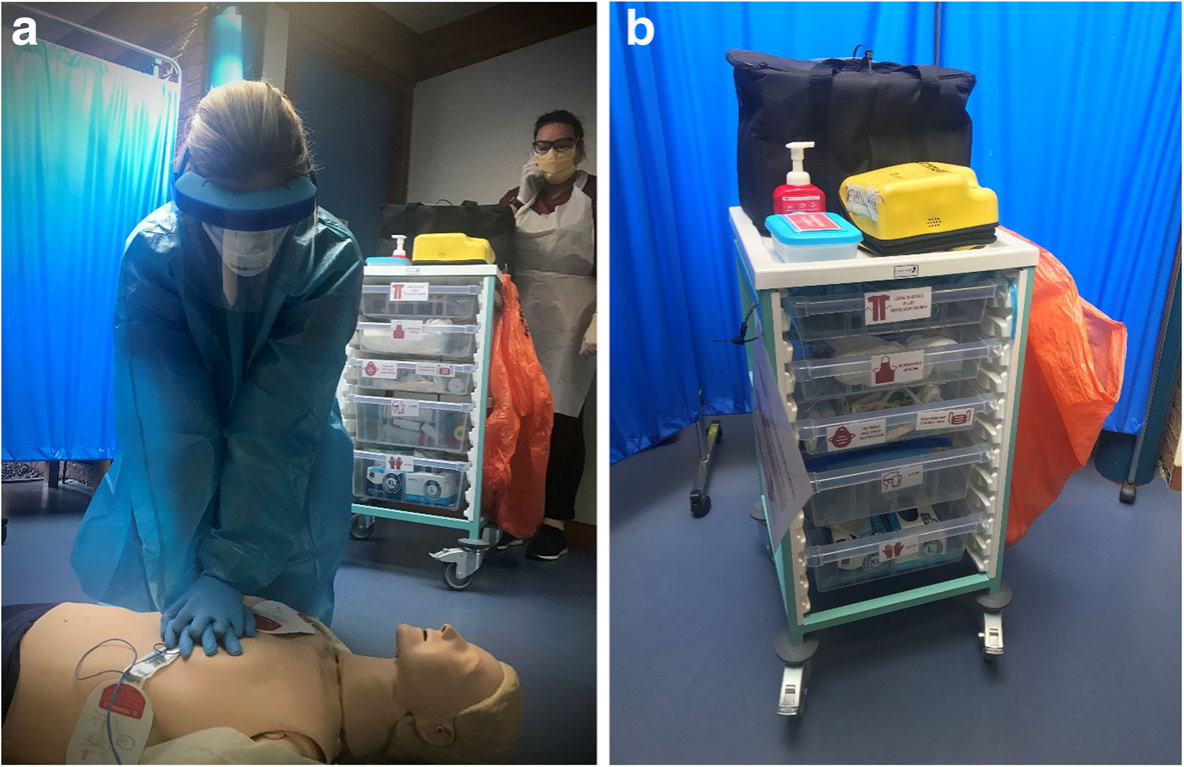
Example of a modified first responder trolley

Tools or artefacts are regarded as essential mediating devices in organizational processes, mediating between the subjects and their objects. In this clinical activity, there are a number of important tools including an AED, defibrillator pads and PPE. As a result of an in situ simulation, it may come to light that the introduction of new tools, or modification of existing tools, may mediate an enhanced response between the *subjects* and the desired *object*. For example during the in situ simulation, a HCW considers starting CPR but only then realizes that they have to don adequate PPE. They then have to go across the building to where the PPE is stored—adding a prolonged delay in commencing CPR. During the debriefing process, CHAT would encourage all to consider this tension and potential solutions. Such an issue is a tension between guidelines *(rules)* recommending the need for enhanced PPE when performing CPR but the PPE *(tools)* is stored in a remote location from a potential casualty (for example PPE being stored in a locker and a casualty in the car park). When a rapid initiation of CPR is desired, *tools* may enable a more efficient process of donning PPE and administering BLS. For example, when carrying out BLS on a patient with COVID-19, it is important that practitioners don adequate PPE efficiently [24]. Through CHAT-guided dialogue during debriefing, it may be suggested that a portable trolley containing the various items of PPE which could confer a beneficial transformation to their system (see Fig. 4). Equally, the PPE could be stored in the sequence of donning, thereby further reducing the time taken to commence BLS. Another advantage of having a portable trolley is that the first responder can take the trolley to where the collapsed individual is, observe the scene and begin to plan their approach (whilst they simultaneously don their PPE).

Whilst all healthcare professionals should be trained in BLS *(rules),* they may not be familiar with the modifications to BLS on patients with COVID-19. This may come to light during the in situ simulation when a HCW realizes they are not update with the most recent guidance on BLS during COVID. Therefore during the debriefing, it may be considered that having a laminated copy *(tool)* of the most recent guidelines *(rules)* could be advantageous and reduce this tension. Equally, having adequate signage *(tools)* to signpost the location of the resuscitation trolley, especially to individuals not familiar with their location (e.g. GP locums *(community)*), could have a significant impact on reactions times to commence BLS. Such examples of change could also be similar to other contexts of utilizing CHAT informed in situ simulation, e.g. in an outpatient department setting or community mental health unit.

- Rules

There are often a multitude of *rules* that govern HCWs’ actions in any clinical environment. Formal *rules* can take the form of higher-level guidelines—for example as is the instance in this case, national resuscitation guidelines [23]—or the recommended procedure of donning/doffing PPE [24]. There can of course be more locally orientated organizational guidelines—including a DNACPR (Do not attempt cardiopulmonary resuscitation) policy or the requirement of healthcare professionals to be certified in BLS. It is important to acknowledge, however, that there can also be ‘informal rules’ applied within an activity system: for example, there may be an implicit understanding that a GP (rather than, for example, a nurse practitioner) takes the lead when providing resuscitative care. Such tacit rules can often be instrumental to the flow within an activity system [25].

As a result of an in situ simulation, a conflict between different guidelines *(rules)* may emerge, for example, a tension between regional and national guidelines on the level of PPE required to carry out BLS—some guidance considers CPR as an aerosol generating procedure and others do not. Therefore, it is important to have a collective resolution of which guidelines to follow through the debriefing process. The in situ simulation may also draw out a tension between the *‘rules’* and the *‘divisions of labour’* in this activity system. It may be considered that GPs normally take the lead when performing BLS on a casualty. However, there may be instances when a GP may not be on the premises (e.g. over lunch time when they are out on house calls)—which might come to light during the debriefing. Therefore during the debriefing process, the practice may consider a policy of conducting regular drills to maintain HCWs skills and all different members of the general practice team (e.g. nurse) to take the lead in performing BLS.

- Community

Beyond the individual who has collapsed and the first responders, it is important to consider the wider *community* that contributes to this clinical activity system. Of course, having a focus on first responders is vital, but there are a number of other individuals and activity systems who contribute to the organizational ability to effectively respond to a collapsed patient. For example, during the in situ simulation, it comes to light that a GP trainee doctor does not know where the AED is stored. Therefore, this raises the importance of administrative staff knowing where the AED is located and ensuring in GP trainee induction *(i.e. rules)*—they are oriented to the practices’ emergency procedures and equipment. In terms of patients’ activity systems, their family members and paramedics and networks make up their *community*. By means of an in situ simulation, perhaps individuals that may have been overlooked in their BLS response may come to light, for example, if other patients of the practice were in close proximity to a collapsed patient and witnessing such an event could be traumatizing for them. Therefore, the dynamic between *community* and *division of labour* may come to light during the debriefing and necessitate the need for a member of staff to marshal the area and liaise with family if they present. As a result of this tension, it may also become evident that a new *tool* may be required—for example, a screen to act as a visual barrier if a patient collapsed in an open space (e.g. the car park).

- Division of labour

There are a number of key tasks that need to take place when responding to a collapsed patient. Dependent on the number of individuals present, and their prior training, allocating key roles ensures that there is a collective and coordinated response—for example, raising the alarm and calling for paramedics, donning PPE, commencing CPR and applying an AED. In addition to the first responders carrying out CPR and applying an AED, there are many other important tasks that may be brought to attention as a result of the in situ simulation. For example, the dynamic between having an individual wait (*division of labour*) for the arrival of a paramedic (the wider *community*) and direct them to the location of the collapsed individual. Another example that could come out of the in situ simulation process is that in order to make the donning process more efficient, and in keeping with guidelines *(rules)*, having a ‘donning buddy’ *(division of labour)* to assist in this task could be beneficial in their transformed system. Again, such tensions and adaptions could equally be similar to other contexts for example community dental practices or pharmacies.

## Conclusion

Often activity theory is utilized as an analytical framework in researching real-life work practices. However, in this paper, we set out how CHAT has potential to provide a theoretical lens and approach to inform in situ simulation to bring about organizational transformation. CHAT enables us to acknowledge the wider system, its underlying tensions and the many contextual factors that influence it. Moreover, it can help to guide the transformative agency of health care professionals, in a collective manner, to bring about change within an entire organizational system [26], in so doing, assisting in the harmonization of ‘work as imagined’ and ‘work as done’ in real authentic clinical environments [27]. Through an activity-theoretical lens, in situ simulation processes may be seen as potentially aiding participants to move beyond their routinized and ‘stabilization’ knowledge, to create ‘possibility knowledge’, enhancing their responsiveness to future activity including unexpected phenomena. Further, it may be interpreted as a micro cycle of collective learning, which may not necessarily lead to expansive learning but involve the potential for it [28]. In sum, given its focus on systems and their interconnections in response to complex phenomena, CHAT has the potential of making the *invisible visible* through in situ simulation and provides insights relevant for health care development beyond the specific simulation context. From this position, we believe that CHAT has the potential to provide in situ simulation with a theoretical framework to optimize organizational development in the diverse contexts of our healthcare systems. We call for empirical based studies that deepen our knowledge of how CHAT can be utilized in guiding in situ simulation in healthcare.

## Data Availability

Not applicable

## Abbreviations

HCWs: Healthcare workers
PPE: Personal protective equipment
CHAT: Cultural-historical activity theory
BLS: Basic life support
AEDs: Automated External Defibrillators
DNACPR: Do not attempt cardiopulmonary resuscitation
